# Visceral Adiposity and Renal Health: From Pathophysiological Mechanisms to Novel Therapeutic Strategies

**DOI:** 10.3390/jcm15187201

**Published:** 2026-09-16

**Authors:** María Marques Vidas, Borja Quiroga, Jose Portoles, Alberto Ortiz, Beatriz Fernández-Fernández, Ana Sánchez Horrillo, María José Soler, Clara García-Carro, Enrique Morales, María A. Bajo

**Affiliations:** 1 Department of Medicine, Universidad Autónoma de Madrid, 28049 Madrid, Spain; 2 Department of Nephrology, Hospital Universitario Puerta de Hierro-Majadahonda, Instituto de Investigación Sanitaria Puerta de Hierro-Segovia de Arana (IDIPHISA), 28222 Madrid, Spain; 3 RICORS2040-Renal (RD24/0004), Instituto de Salud Carlos III, 28029 Madrid, Spainmjsoler01@gmail.com (M.J.S.); mauxiliadora.bajo@salud.madrid.org (M.A.B.); 4 Department of Nephrology, Hospital Universitario de la Princesa, Instituto de Investigación Sanitaria del Hospital Universitario de La Princesa (IIS Princesa), 28006 Madrid, Spain; 5 Department of Nephrology and Hypertension, Instituto de Investigación Sanitaria Fundación Jiménez Díaz (IIS-FJD), Hospital Universitario Fundación Jiménez Díaz, 28040 Madrid, Spain; 6 Department of Nephrology, Hospital Universitario Vall d’Hebron, Centro de Referencia en Enfermedad Glomerular Compleja del Sistema Nacional de Salud (CSUR), Vall d’Hebron Institut de Recerca (VHIR), 08035 Barcelona, Spain; 7 Department of Nephrology, San Carlos University Clinical Hospital, Instituto de Investigación Sanitaria del Hospital Clínico San Carlos (IdISSC), 28040 Madrid, Spain; 8 Department of Nephrology, Hospital Universitario 12 de Octubre, 28041 Madrid, Spain; emoralesr@senefro.org

**Keywords:** chronic kidney disease, obesity, progression, visceral adipose tissue

## Abstract

Obesity-related chronic kidney disease (ob-CKD) is an increasingly prevalent condition whose pathogenesis extends beyond excess body mass to the metabolic dysfunction of visceral adipose tissue (VAT). This narrative review synthesizes the mechanistic, diagnostic, and therapeutic dimensions of the adiporenal axis: the bidirectional crosstalk between dysfunctional visceral fat and the kidney. From a pathophysiological perspective, VAT promotes renal injury through four converging pathways: hemodynamic overload via the renin–angiotensin–aldosterone system and sympathetic activation; adipokine imbalance (leptin excess/adiponectin deficiency); chronic inflammation mediated by tumor necrosis factor-α (TNF-α) and interleukin-6 (IL-6); and direct lipotoxicity from ectopic renal fat deposition. Because body mass index (BMI)fails to capture these pathogenic mechanisms, visceral adiposity-centered assessment (using waist circumference, the visceral adiposity index, and cross-sectional imaging) is essential for accurate risk stratification. The recently proposed ob-CKD classification (types 1–5) links disease stage to therapeutic strategy across the full CKD continuum. The therapeutic landscape now includes agents with combined weight-loss and nephroprotective properties: glucagon-like peptide-1 (GLP-1) receptor agonists (FLOW trial), dual glucose-dependent insulinotropic polypeptide (GIP)/GLP-1 agonists (tirzepatide), sodium-glucose co-transporter 2 inhibitors (SGLT2i), and non-steroidal mineralocorticoid receptor antagonists (finerenone), alongside metabolic surgery for refractory cases. This review provides an integrative framework for shifting clinical practice from BMI-centric to visceral adiposity-driven approaches in the prevention and management of ob-CKD.

## 1. Introduction

Obesity has become a major global driver of CKD, but its renal impact is determined less by total body mass than by the biological dysfunction of VAT. Traditional metrics illustrate a progressive refinement in risk capture: BMI, the simplest tool, conflates lean mass with fat and ignores distribution; waist circumference better reflects central fat accumulation and predicts albuminuria even in individuals with BMI < 25 kg/m^2^ [1]. However, only VAT assessment identifies the pathogenic compartment: the inflamed, metabolically dysregulated depot whose secretory products directly engage the kidney through what has been termed the adiporenal axis [2,3]. This progression from BMI to waist circumference to VAT parallels a deeper conceptual shift: from adiposity as an anthropometric trait to adiposopathy, the recognition that tissue dysfunction, not mass alone, underlies cardiometabolic and renal injury [4].

Ob-CKD encompasses the structural and functional renal alterations driven by hemodynamic overload, chronic low-grade inflammation, oxidative stress, and metabolic derangements associated with excess visceral adiposity [5]. Epidemiological data indicate that obesity has become one of the fastest-growing contributors to CKD worldwide [6], and Mendelian randomization supports a causal, not merely associative, link between genetically predicted VAT and CKD risk [2]. Despite this evidence, VAT assessment remains underutilized in clinical practice, leading to persistent risk misclassification. This review synthesizes the pathways through which VAT promotes renal injury, establishes the biological framework of the adiporenal axis, and evaluates how this framework informs clinical assessment and emerging therapeutic strategies.

For clarity, several related but distinct terms are used throughout this review. ‘Obesity-related CKD’ (ob-CKD) is an umbrella term encompassing the full spectrum of renal structural and functional alterations caused or exacerbated by excess adiposity, as defined by the recent SEN-SLANH-SEEDO consensus [5]. ‘Obesity-related glomerulopathy’ (ORG) refers specifically to the histopathological entity characterized by glomerulomegaly with or without secondary focal segmental glomerulosclerosis (ob-CKD type 2). The ‘adiporenal axis’ describes the bidirectional pathophysiological crosstalk between dysfunctional visceral adipose tissue and the kidney, representing the mechanistic framework underlying ob-CKD. ‘Fatty kidney’ refers to ectopic lipid deposition within renal parenchymal cells, a contributor to lipotoxic injury but not synonymous with ORG or ob-CKD as a whole.

For this narrative review, we searched PubMed, Scopus, and Web of Science from inception through June 2026, using the terms ‘visceral adiposity,’ ‘obesity-related kidney disease,’ ‘obesity-related glomerulopathy,’ ‘adiporenal,’ ‘renal lipotoxicity,’ and individual drug class names combined with ‘kidney.’ Reference lists of selected articles and recent systematic reviews were also screened. No formal systematic search protocol or risk-of-bias assessment was applied. Throughout the manuscript, we distinguish findings derived from preclinical or mechanistic models from those supported by human observational data or randomized controlled trials.

## 2. Pathophysiological Mechanisms of VAT-Induced Renal Damage

**Figure 1:**
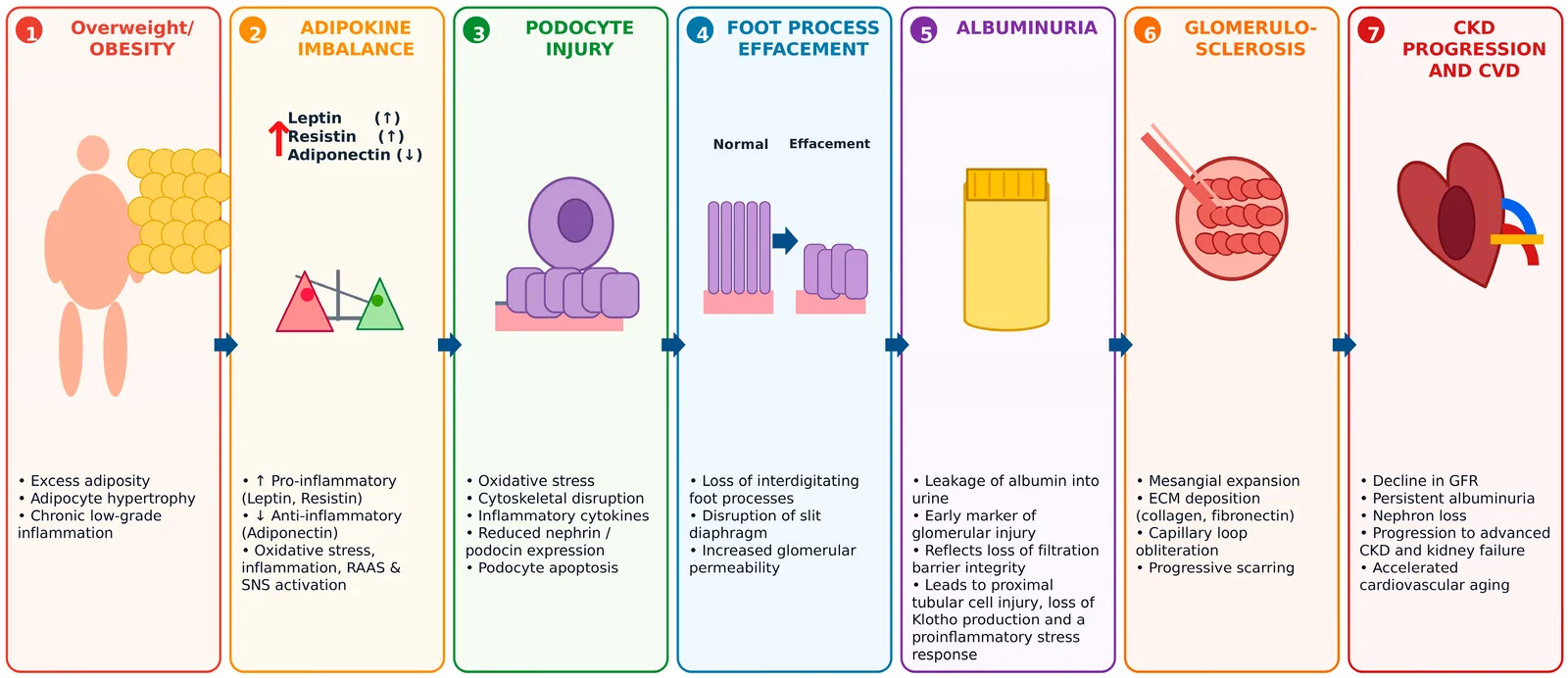
Stepwise cascade from visceral adiposity to chronic kidney disease. Schematic representation of the seven-step pathophysiological cascade linking visceral adipose tissue accumulation to CKD. The sequence encompasses: (**1**) visceral fat expansion, (**2**) adipokine dysregulation and inflammatory signaling, (**3**) hemodynamic stress (RAAS activation, sympathetic overactivity), (**4**) glomerular hyperfiltration and hypertension, (**5**) podocyte injury and proteinuria, (**6**) tubulointerstitial inflammation and fibrosis, and (**7**) progressive nephron loss leading to established CKD. Abbreviations: CKD: chronic kidney disease; GFR: glomerular filtration rate; RAAS: renin–angiotensin–aldosterone system; SNS: sympathetic nervous system. Arrows indicate the direction of change: ↑ increased; ↓ decreased.

The pathophysiological mechanisms of VAT-induced renal damage are multipronged. They include hemodynamic alterations, adipokine dysregulation, systemic inflammation and oxidative stress, and lipotoxicity and ectopic fat deposition (Figure 1).

### 2.1. Neurohormonal and Hemodynamic Alterations

**Figure 2:**
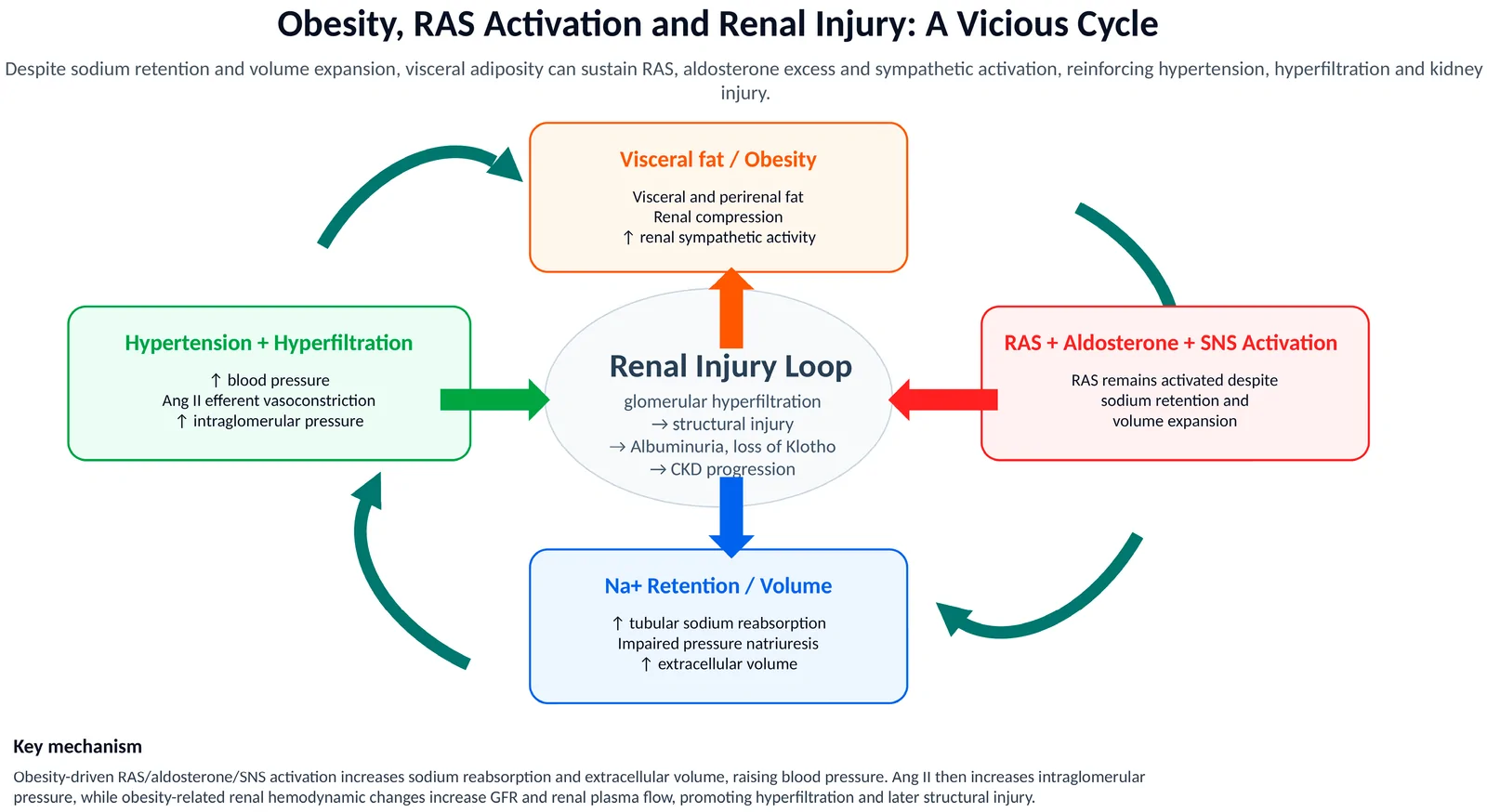
Vicious cycle of renin–angiotensin–aldosterone system (RAAS) activation in obesity-related kidney disease. This figure illustrates how excess visceral adipose tissue activates the intrarenal and systemic RAAS, leading to angiotensin II-mediated efferent arteriolar vasoconstriction, aldosterone excess, sympathetic nervous system overactivation, and sodium retention. These hemodynamic disturbances converge on glomerular hypertension and hyperfiltration, which drive podocyte injury, proteinuria, and progressive glomerulosclerosis, establishing a self-perpetuating cycle of renal damage. Abbreviations: Ang II: angiotensin II; CKD: chronic kidney disease; GFR: glomerular filtration rate; RAS: renin–angiotensin system; SNS: sympathetic nervous system. ↑ indicates an increase; → indicates a causal or sequential step in the cycle. Based on [10,12,14,27,28].

Obesity triggers a neurohormonal cascade from adipose and intrarenal renin–angiotensin system (RAS) activation to aldosterone excess and sympathetic overactivity, causing glomerular hypertension and single-nephron hyperfiltration [7,8,9,10] (Figure 2). Glomerular hyperfiltration is now recognized as the main driver of CKD progression, as it is decreased by all current nephron-sparing drugs [11]. The resulting glomerular hypertension directly injures podocytes, causes podocyte loss, glomerulosclerosis, and increases albumin filtration [12,13]. Excess filtered albumin overwhelms proximal tubular reabsorption capacity, producing proteotoxicity, loss of gerosuppressor functions (Klotho), and an inflammatory response that magnifies kidney injury [14,15]. Adipose tissue produces angiotensinogen, aldosterone, and inflammatory mediators [16,17], and excess adiposity chronically activates both the RAS and the sympathetic nervous system through an adipose–brain–kidney axis [18]. Sympathetic overactivity promotes vasoconstriction, sodium retention, and hypertension [19,20], while insulin resistance further stimulates sodium reabsorption and RAS activation, creating a vicious cycle [12].

Importantly, aldosterone is regulated not only by angiotensin II but also by Klotho deficiency and hyperkalemia, among others [21]; thus, RAS blockade alone may not sufficiently suppress aldosterone, and the addition of a mineralocorticoid receptor antagonist (MRA) may help to further optimize cardiorenal outcomes [22,23]. Aldosterone promotes inflammation, oxidative stress, endothelial dysfunction, and fibrosis, contributing to CKD progression even in the absence of other cardiovascular conditions [9,24]. Although mechanistic and animal studies strongly support adipose-derived RAS and aldosterone as contributors to renal injury [25,26], interventional human data remain limited. Nonetheless, Mendelian randomization supports a causal role of VAT in CKD, with hypertension mediating approximately 46% of this effect [2], and perirenal fat correlates with decreased estimated glomerular filtration rate (eGFR) and CKD burden in patients with resistant hypertension [27].

### 2.2. Adipokine Dysregulation

Adipose tissue acts as an endocrine organ secreting adipokines that regulate metabolism, inflammation, and vascular function. In obesity, adipocyte hypertrophy shifts this balance toward a pro-inflammatory and pro-fibrotic profile characterized by increased leptin and resistin and reduced adiponectin, alongside elevated cytokines such as TNF-α, IL-6, and monocyte chemotactic protein-1 (MCP-1) [12,17]. Leptin, physiologically involved in appetite regulation via the hypothalamic POMC-MC4R pathway [29,30], increases proportionally with fat mass and is elevated in CKD. In experimental models, leptin stimulates transforming growth factor-beta 1 (TGF-β1) production, extracellular matrix deposition, mesangial proliferation, sympathetic activation, and adrenal aldosterone secretion [12,31], and directly impairs podocyte homeostasis [31,32]. Resistin, predominantly secreted by macrophages in humans, stimulates proinflammatory cytokine production and reduces endothelial nitric oxide (NO) availability [33,34]. In contrast, adiponectin, which decreases as visceral adiposity increases, preserves podocyte structure by maintaining nephrin and podocin expression through AdipoR1/R2 activation [35,36].

Beyond classical adipokines, additional adipose-derived mediators (autotaxin, platelet-derived growth factor D, lipocalin-2, fatty acid binding proteins, lipoxygenases) have been implicated in experimental models of obesity-related renal and cardiovascular injury within the cardiovascular–kidney–metabolic syndrome [37]. Targeted suppression of these factors within adipose tissue mitigates inflammation, fibrosis, insulin resistance, and organ damage in murine studies [26]. Despite robust mechanistic and preclinical evidence, human interventional data confirming causal relationships between adipokine dysregulation and kidney injury remain scarce.

### 2.3. Inflammation and Oxidative Stress

**Figure 3:**
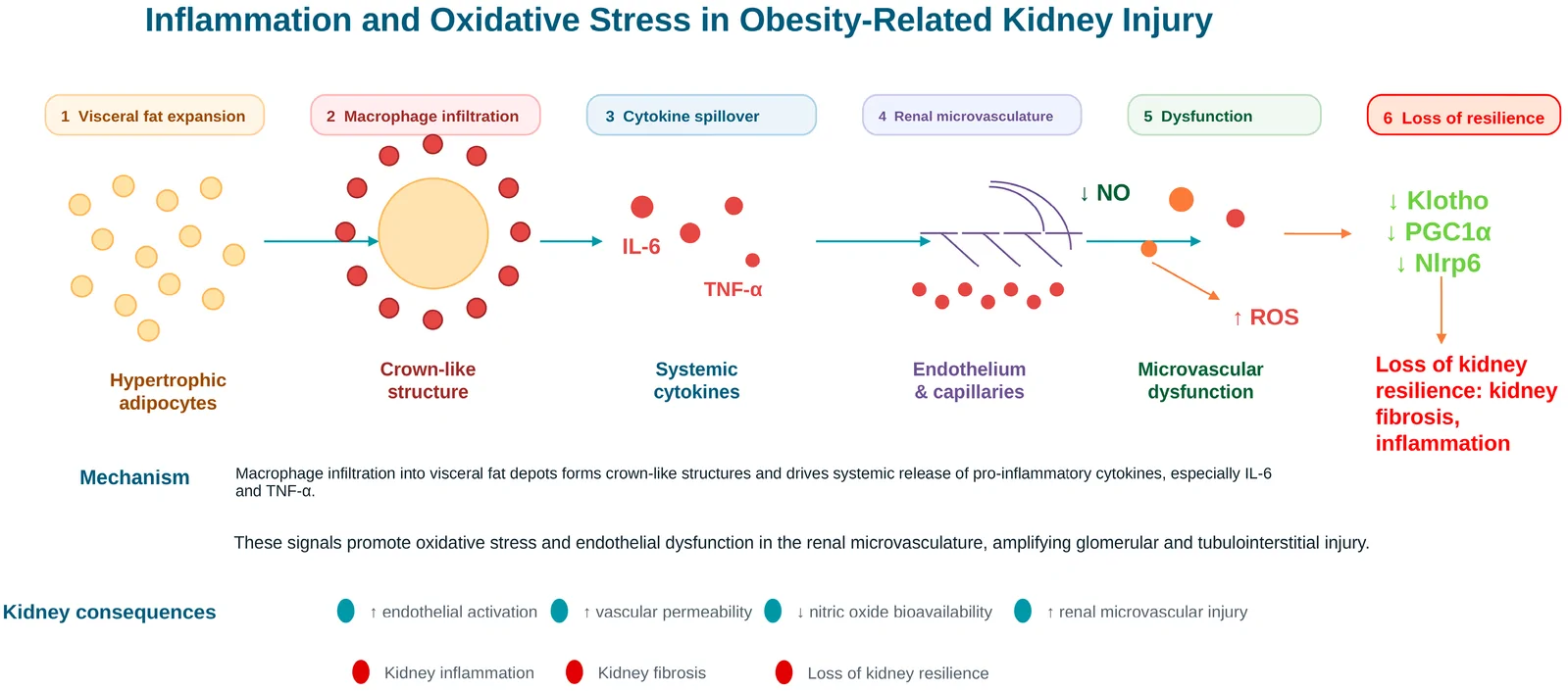
Inflammatory and oxidative stress pathways in visceral adiposity-driven kidney injury. Overview of the molecular mechanisms by which inflamed visceral and perirenal adipose tissue promotes renal damage. As demonstrated in human adipose tissue biopsies and animal models, hypertrophic adipocytes and crown-like structures release pro-inflammatory cytokines (TNF-α, IL-6) that activate NF-κB and JAK/STAT signaling, stimulate NADPH oxidase-dependent reactive oxygen species generation, and impair endothelial nitric oxide bioavailability. These processes lead to glomerular endothelial dysfunction, podocyte injury, tubular stress, and progressive interstitial fibrosis. The figure also highlights the paracrine effects of perirenal fat and the downregulation of kidney-protective factors (Klotho, PGC1α, NLRP6). Abbreviations: IL-6: interleukin-6; NLRP6: NLR family pyrin domain containing 6; NO: nitric oxide; PGC1α: peroxisome proliferator-activated receptor gamma coactivator 1-alpha; ROS: reactive oxygen species; TNF-α: tumor necrosis factor alpha. ↑ increased; ↓ decreased.

Expansion and dysfunction of visceral and perirenal adipose tissue promote macrophage activation and release of TNF-α and IL-6 (Figure 3), both of which cause kidney and cardiovascular injury [38,39]. Although TNF-α has been implicated in kidney injury since the 1990s [40], no anti-TNF-α therapy is currently approved for kidney protection. Clinical trials assessing anti-IL-6 therapies for CKD are ongoing [41,42]. In phase 2 trials, targeting IL-6 reduced lipoprotein(a), improved anemia control, and decreased systemic inflammation. Inflammatory cytokines amplify oxidative stress, promote endothelial activation and dysfunction, and reduce nitric oxide bioavailability, contributing to microvascular dysfunction, podocyte injury, and fibrosis [13,43]. A characteristic histological feature is the crown-like structure, in which macrophages aggregate around dying hypertrophic adipocytes and release inflammatory mediators [44]; visceral fat exhibits stronger pro-inflammatory macrophage phenotypes than subcutaneous fat [45].

TNF-α and IL-6 activate NF-κB and JAK/STAT signaling, stimulate reactive oxygen species generation through NADPH oxidases and mitochondrial dysfunction, and impair endothelial nitric oxide synthase activation, leading to endothelial dysfunction [46,47]. Human obesity is associated with microvascular endothelial dysfunction driven by enhanced oxidative stress [48,49], while systemic cytokines increase endothelin-mediated vasoconstriction and leukocyte adhesion, damaging glomerular endothelium and favoring albuminuria [50]. The peritubular microcirculation is also affected, promoting local hypoxia, tubular stress, and interstitial fibrosis [28,49]. Notably, GLP-1 receptor agonists (GLP-1 RAs) exert kidney-protective effects partly through modulation of glomerular endothelial cell biology [51,52]. Systemic inflammation additionally decreases kidney resilience through downregulation of protective factors such as Klotho, PGC1α, and Nlrp6 [15,53]. Perirenal adipose tissue can also release cytokines and vasoactive factors in close proximity to the renal vasculature, exerting local paracrine effects [9,13,24]. Notably, several nephroprotective agents discussed in Section 5, including SGLT2i, GLP-1 RAs, and non-steroidal MRAs, also exert anti-inflammatory effects that may contribute to their renal benefits beyond their primary mechanisms of action.

### 2.4. Ectopic Fat Deposition and Lipotoxicity

Renal sinus and perirenal fat accumulation damage the kidney through mechanical compression and lipotoxicity. Mechanical compression disrupts renal hemodynamics by increasing intrarenal/interstitial pressure, reducing vasa recta blood flow, impairing natriuresis, and activating the RAS [43]. In dogs and humans with obesity, this compression increases extracellular matrix deposition, displaces renal parenchyma, and contributes to an enlarged, rounded kidney [12,54]. Reduced renal blood flow activates the RAS, which further increases intrarenal venous pressure and promotes sodium reabsorption, creating a self-perpetuating cycle.

In preclinical studies, ectopic fatty acid uptake by podocytes and proximal tubular cells promotes lipid droplet accumulation, mitochondrial dysfunction, ROS generation, NLRP3 activation, and fibrosis [43]. Imbalanced fatty acid uptake and oxidation leads to accumulation of toxic lipid metabolites such as ceramides and diacylglycerols [55]. In proximal tubular cells, CD36/KIM-1-mediated fatty acid uptake impairs mitochondrial function and promotes inflammation and fibrosis [56,57]. KIM-1 is itself an injury marker, creating a vicious circle in which injury facilitates fatty acid uptake and fatty acid uptake causes further injury. Circulating fatty acids also accumulate as ectopic lipid droplets in podocytes, inducing cytoskeletal damage and apoptosis [43,55].

## 3. Clinical Assessment: Moving Beyond BMI

BMI is the most widely used metric to classify obesity in nephrology practice, yet it conflates lean mass, bone, and fat, and is agnostic to fat distribution. These limitations are amplified in CKD, where sarcopenia, fluid overload, and muscle wasting distort the weight-adiposity relationship and produce the “obesity paradox” [40]. Accurately staging ob-CKD therefore requires measures that identify the pathogenic visceral compartment.

### 3.1. Imaging Modalities

**Figure 4:**
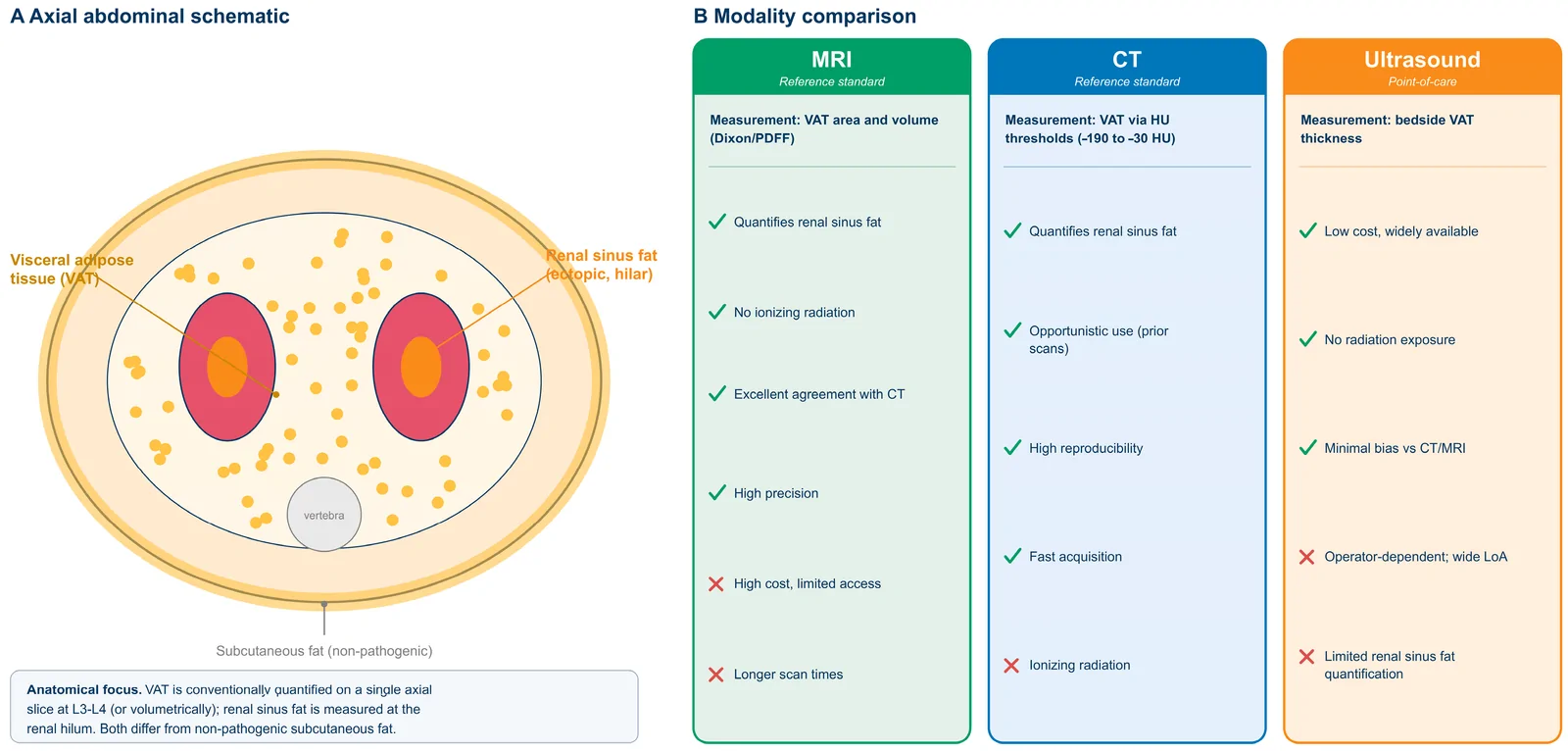
Imaging modalities for visceral and renal fat quantification. Comparative overview of the imaging techniques available for quantifying visceral adipose tissue and renal sinus fat, including computed tomography (CT), magnetic resonance imaging (MRI), ultrasound (US), dual-energy X-ray absorptiometry (DXA), and bioelectrical impedance analysis (BIA). For each modality, the figure presents the measurement principle, anatomical targets, key advantages and limitations, and clinical applicability for risk stratification in obesity-related kidney disease. Abbreviations: CT: computed tomography; PDFF: proton-density fat fraction; VAT: visceral adipose tissue. HU: Hounsfield units; LoA: limits of agreement. ✓ indicates an advantage or strength of the modality; ✗ indicates a limitation.

Clinical assessment of adiposity has evolved along a trajectory of increasing specificity: BMI captures total mass but ignores distribution; waist circumference reflects central fat but cannot distinguish visceral from subcutaneous depots; and cross-sectional imaging directly quantifies VAT, the depot most strongly linked to renal injury. Computed tomography (CT) segments VAT using fat-specific attenuation thresholds (approximately −190 to −30 HU) on single axial slices at L3-L4 or across volumetric stacks. Magnetic resonance imaging (MRI) achieves comparable quantification without ionising radiation using Dixon or proton-density fat-fraction sequences, and shows close agreement with CT. [58]. Importantly, imaging also enables quantification of renal sinus fat, an ectopic depot invisible to anthropometric surrogates; higher renal sinus fat volumes are associated with hypertension and future >50% eGFR decline in biopsied CKD patients, making it a promising kidney-specific biomarker [3] (Figure 4).

The principal drawback of CT and MRI is limited feasibility for routine screening: CT entails radiation, MRI is constrained by cost and access, and dedicated scans solely for adiposity are rarely justified. Opportunistic analysis of abdominal CT obtained for other indications, increasingly aided by automated and deep-learning tools, provides a pragmatic solution. Ultrasound offers a genuine point-of-care alternative (inexpensive, radiation-free, and bedside) measuring visceral fat thickness as a surrogate; meta-analyses show minimal systematic bias versus CT/MRI, although operator dependence widens limits of agreement [58]. Separately, bioelectrical impedance analysis consistently underestimates visceral fat compared with CT/MRI [58] and is not recommended as a primary assessment tool. Across all modalities, heterogeneous acquisition protocols and the absence of standardized, kidney-specific cut-offs still limit clinical translation.

### 3.2. Clinical Surrogate Markers

Where imaging is unavailable, anthropometric and composite indices provide accessible estimates of central adiposity. Waist circumference predicts albuminuria even in individuals with BMI < 25 kg/m^2^ and is endorsed within the cardiovascular–kidney–metabolic framework [1], though it cannot separate visceral from subcutaneous fat. The waist-to-hip ratio (WHR) partially addresses this; in the MESA cohort, WHR was associated with rapid kidney-function decline [59], and in meta-analyses of CKD patients, high WHR predicted increased mortality even as higher BMI appeared paradoxically protective [60,61]. Composite indices such as the Visceral Adiposity Index (VAI) and lipid accumulation product (LAP) combine anthropometry with serum triglycerides and HDL cholesterol. In a population-based cohort, ascending VAI quartiles independently predicted incident albuminuria and CKD [62], although in the REGARDS cohort VAI and LAP did not add predictive information beyond waist circumference and BMI [63]. These surrogates are attractive for scalability but lack ethnicity-specific cut-offs (Section 6) and may miss sarcopenic obesity; pairing a distribution-sensitive marker with an estimate of lean mass is preferable [43,58].

### 3.3. Epidemiological Evidence

The clinical case for visceral-fat-centered assessment rests on consistent cohort and genetic evidence (Table 1). Mendelian randomization using UK Biobank and CKDGen/FinnGen data supports a causal effect of genetically predicted VAT on CKD risk (odds ratios 1.18–1.47), with hypertension mediating roughly half the effect [2]. At the tissue level, renal sinus fat predicts >50% eGFR loss [3], while VAI and LAP predict incident albuminuria and CKD across diverse populations [62,63,64]. Across study designs, distribution-sensitive markers are more consistently associated with renal outcomes than BMI, and the obesity paradox in advanced CKD largely reflects BMI’s inability to separate protective lean mass from harmful visceral fat [60,61]. Clinically, these data support a tiered approach: standardized waist circumference and VAI/LAP at minimum, opportunistic VAT and renal sinus fat quantification when imaging is available, and dedicated MRI for research or selected patients requiring serial monitoring. This BMI-to-waist-circumference (WC)-to-imaging hierarchy underscores a central message: progressing from global to distribution-sensitive to depot-specific assessment progressively improves risk capture and should guide the tiered evaluation of every patient with obesity and CKD.

## 4. Clinical Implications: Obesity-Related Chronic Kidney Disease (ob-CKD)

Ob-CKD may be recognized by clinical and histopathological features and prognostic indicators that support an integrated clinical perspective.

### 4.1. Clinical Presentation

Ob-CKD encompasses a spectrum of functional and structural renal alterations driven by the interplay between obesity, hemodynamic stress, metabolic dysfunction, and chronic inflammation [13]. According to the SEN-SLANH-SEEDO consensus classification (Table 2), it should be noted that this classification represents an expert-consensus proposal and has not been formally endorsed by KDIGO or other international guideline bodies; prospective validation studies are warranted. ob-CKD type 1 corresponds to reversible functional abnormalities, while type 2 (corresponding to obesity-related glomerulopathy (ORG)) is characterized by persistent subnephrotic proteinuria with bland sediment, preserved renal function, and absence of alternative etiologies [5]. Full nephrotic syndrome is uncommon (<5%); structural injury including glomerulomegaly and early FSGS may be present even in normoalbuminuric individuals, thus preceding the albuminuric stage [6,66]. The clinical trajectory is one of slowly progressive proteinuria with variable rates of eGFR decline, commonly associated with cardiometabolic comorbidities that integrate ob-CKD within the cardiovascular–kidney–metabolic syndrome (type 3) [67]. Metabolically unhealthy phenotypes, characterized by visceral adiposity, insulin resistance, and a pro-inflammatory milieu, confer substantially higher risk [16].

Once patients reach kidney failure requiring kidney replacement therapy (type 4), the obesity paradox becomes apparent, likely reflecting the confounding effect of malnutrition [61,68]. In the post-transplant setting (type 5), obesity is associated with higher surgical complications, infections, delayed graft function, and reduced access to transplantation [5,69].

### 4.2. Renal Biopsy Findings and Histopathological Features

**Figure 5:**
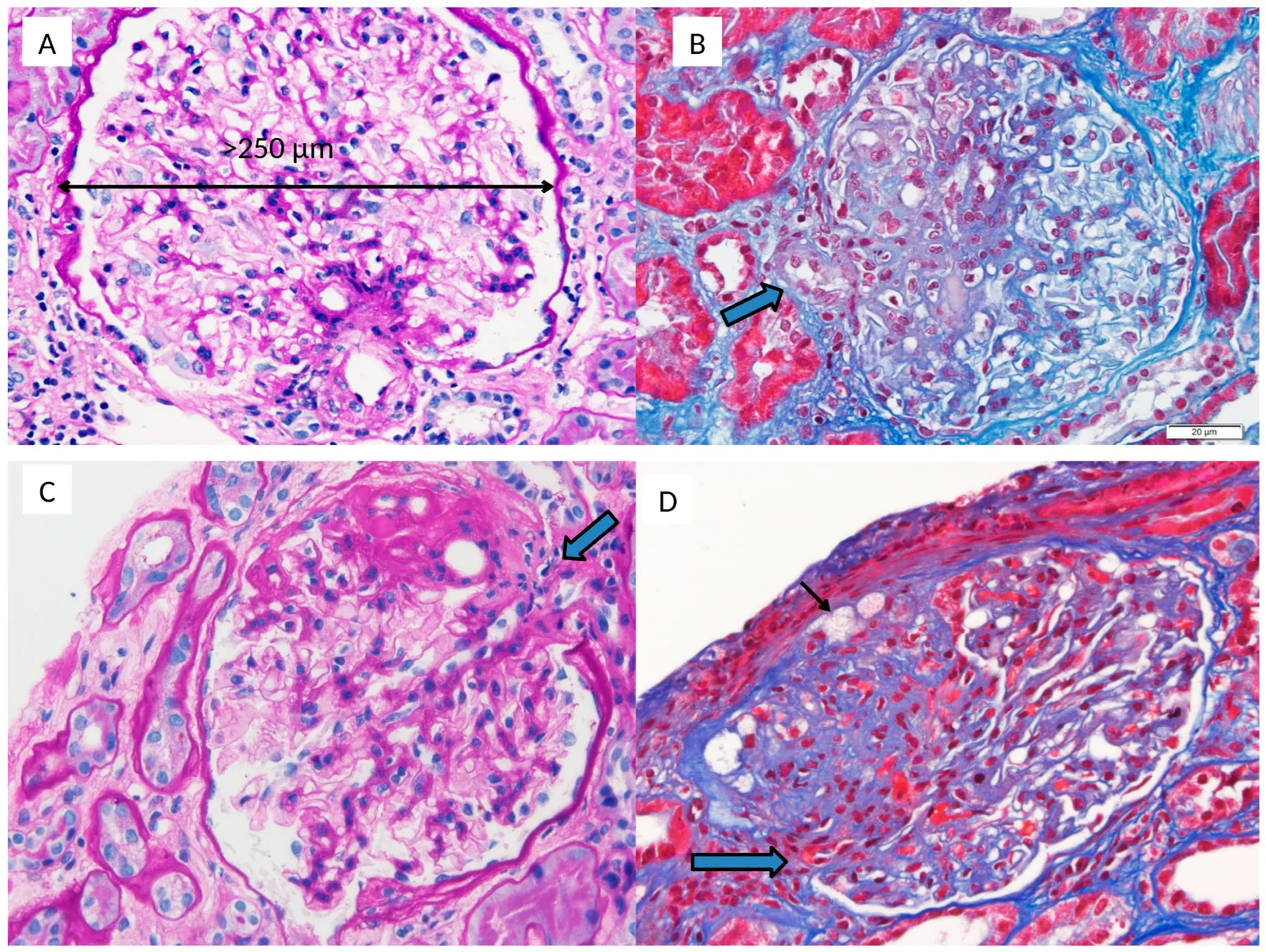
Histopathological features of obesity-related glomerulopathy (ORG). Representative renal biopsy findings. (**A**) Glomerulomegaly: the double-headed arrow indicates a maximal glomerular diameter exceeding 250 µm (periodic acid–Schiff). (**B**) Secondary focal segmental glomerulosclerosis with a perihilar distribution; the arrow indicates the segmental lesion (Masson trichrome). (**C**) Secondary focal segmental glomerulosclerosis with a perihilar distribution; the arrow indicates the segmental lesion (periodic acid–Schiff). (**D**) Foam cells with optically empty, finely vacuolated cytoplasm (thin arrow), consistent with lipid dissolved during routine processing, adjacent to an area of segmental sclerosis (block arrow) (Masson trichrome). Abbreviations: FSGS, focal segmental glomerulosclerosis; ORG, obesity-related glomerulopathy; PAS, periodic acid–Schiff.

When performed, renal biopsy provides the most definitive diagnostic information, revealing chronic exposure to glomerular hyperfiltration and metabolic stress (Figure 5). The hallmark lesion is glomerulomegaly, reflecting adaptive hypertrophy that ultimately becomes maladaptive. Secondary FSGS, typically perihilar, follows a more indolent course than primary FSGS (Table 3). Podocyte injury is central to progression and is characterized by hypertrophy, cytoskeletal stress, and progressive depletion, with foot process effacement that is typically mild and segmental (<50%) [24,70]. Ectopic lipid accumulation within podocytes, mesangial cells, and proximal tubular cells (the “fatty kidney”) contributes to progression through lipotoxicity [43,55]. Additional features include mesangial expansion with mild “diabetoid” changes, tubulointerstitial fibrosis, and arteriolar hyalinosis. Immunofluorescence is typically nonspecific [71,72]. Importantly, ORG often coexists with other renal pathologies within ob-CKD type 3, underscoring the role of biopsy in identifying overlapping lesions [5,73]. It should be noted that no single histological feature is pathognomonic for ob-CKD; the diagnosis relies on a characteristic pattern (glomerulomegaly, secondary FSGS, podocyte depletion) in the appropriate clinical context, after exclusion of alternative etiologies. Although ectopic lipid accumulation (“fatty kidney”) is pathophysiologically relevant, its systematic histological evaluation is limited by the dissolution of lipid during conventional tissue processing, and standardized criteria or cut-offs for renal lipid content have not been established.

### 4.3. Silent Progression and Prognostic Indicators

Despite its apparently benign initial presentation, ob-CKD is progressive, with 10–33% of patients advancing to advanced CKD or kidney failure [66,74]. As for other kidney diseases, proteinuria magnitude and persistence are the strongest predictors of outcome; additional determinants include baseline renal function, obesity duration, histological chronicity, and metabolic factors (insulin resistance, hypertension, dyslipidemia) [75]. Emerging evidence underscores adipose tissue dysfunction and ectopic lipid deposition as key drivers, while organ-specific fat depots (renal sinus, perirenal) exacerbate injury through mechanical compression and local inflammation. Reduced nephron endowment further increases susceptibility to hyperfiltration [76]. A particularly important clinical concept is the silent nature of progression: conventional biomarkers lack sensitivity for early structural damage, and despite therapeutic choices including RAS blockade, SGLT2i, and incretins, a significant proportion of patients continue to progress [77].

### 4.4. Integrated Clinical Perspective

Ob-CKD should not be considered an isolated glomerular entity but rather a dynamic, potentially modifiable continuum of metabolic-inflammatory nephropathy in which adaptive responses to increased functional demand ultimately become maladaptive [6]. The recognition of the “fatty kidney” phenotype and the role of dysfunctional adipose tissue as a source of pro-inflammatory cytokines provides a mechanistic link between systemic metabolic disease and local kidney injury [5,16]. Therapeutic strategies should adopt a comprehensive, multifactorial approach targeting both hemodynamic and metabolic pathways, including optimized RAS blockade (ACEi/ARB), non-steroidal MRAs, SGLT2i, incretin-based therapies, and sustained weight reduction aimed at reducing ectopic fat burden [77].

## 5. Therapeutic Strategies Targeting the AdipoRenal Axis

**Figure 6:**
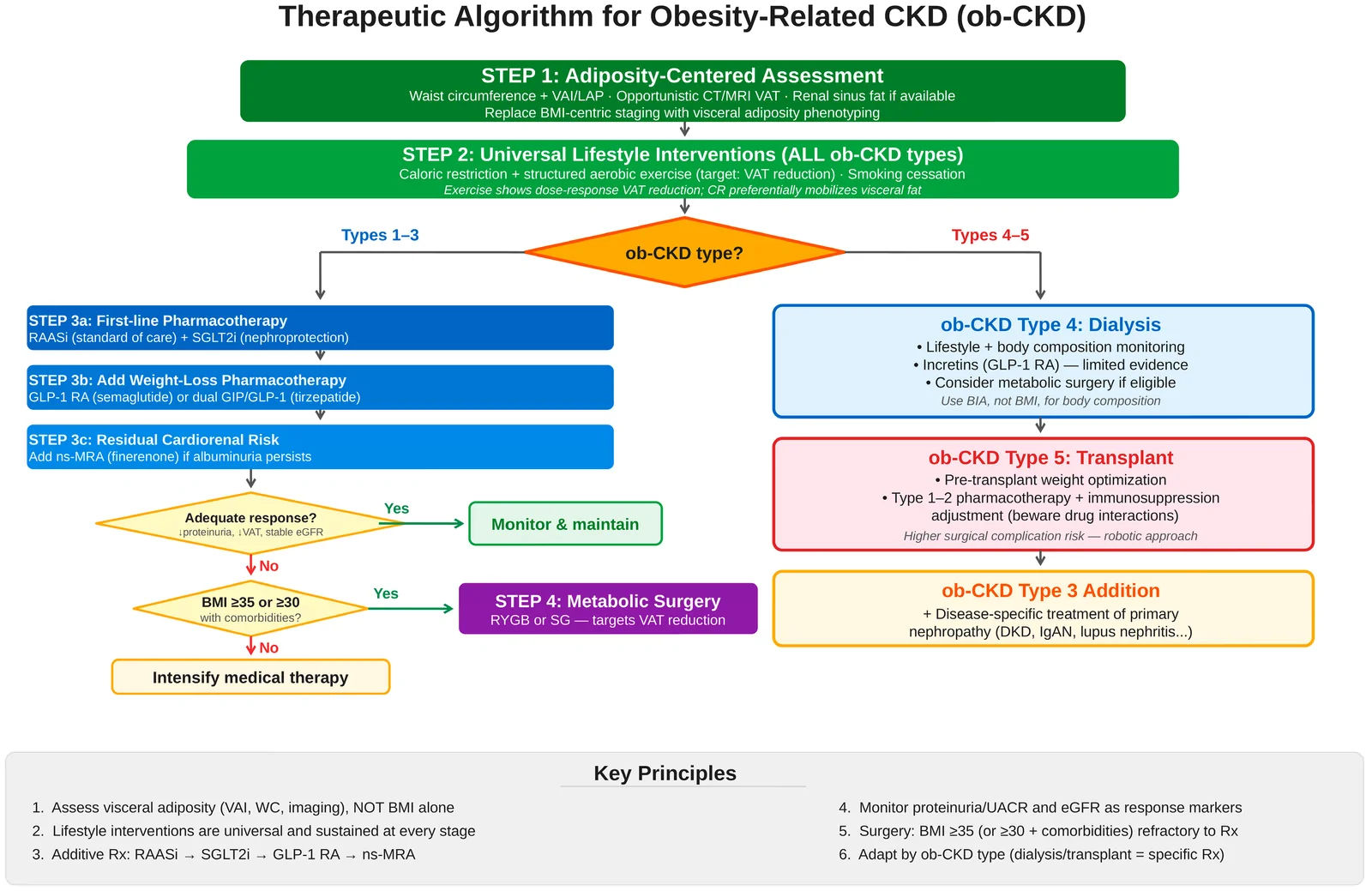
Proposed therapeutic algorithm for the management of obesity-related chronic kidney disease (ob-CKD). Stepwise therapeutic algorithm for the integrated management of obesity-related CKD, stratified by disease stage. The algorithm proposes a tiered approach: (1) universal lifestyle interventions (caloric restriction, structured aerobic exercise targeting visceral adipose tissue reduction) for all patients; (2) first-line pharmacotherapy with SGLT2 inhibitors (hemodynamic nephroprotection) and GLP-1 receptor agonists or dual GIP/GLP-1 agonists (weight loss and metabolic benefits); (3) addition of non-steroidal mineralocorticoid receptor antagonists (finerenone) for residual cardiorenal risk; (4) consideration of bariatric/metabolic surgery for patients with severe obesity (BMI ≥ 35 kg/m^2^) or inadequate response to medical therapy; and (5) stage-specific adaptations for dialysis (ob-CKD type 4) and kidney transplant recipients (ob-CKD type 5). Decision nodes incorporate visceral adiposity assessment (VAI, waist circumference, imaging when available) rather than BMI alone. Note: This algorithm represents a pragmatic expert-opinion framework based on currently available evidence and should not be interpreted as formal guideline recommendations or mandatory clinical guidance. Abbreviations: BMI: body mass index; CKD: chronic kidney disease; eGFR: estimated glomerular filtration rate; GIP: glucose-dependent insulinotropic polypeptide; GLP-1 RA: glucagon-like peptide-1 receptor agonist; MRA: mineralocorticoid receptor antagonist; ns-MRA: non-steroidal mineralocorticoid receptor antagonist; ob-CKD: obesity-related chronic kidney disease; RAASi: renin–angiotensin–aldosterone system inhibitor; SGLT2i: sodium-glucose cotransporter 2 inhibitor; UACR: urinary albumin-to-creatinine ratio; VAI: Visceral Adiposity Index; VAT: visceral adipose tissue. ↓ indicates a reduction in the corresponding parameter.

The recognition of the adipo–renal axis as a pivotal pathophysiological pathway linking obesity and CKD has substantially reshaped therapeutic strategies. Figure 6 shows a therapeutic algorithm categorized by CKD stage, and Table 4 summarizes the key clinical trials evaluating nephroprotection and weight-loss efficacy.

† STEP 1 and SURMOUNT-1 are included to contextualize the magnitude of weight reduction achievable with incretin-based therapies, which is directly relevant to visceral adipose tissue reduction in ob-CKD. The FLOW trial subsequently demonstrated kidney-specific benefits for semaglutide, and the TREASURE-CKD trial (NCT05536804) is evaluating renal outcomes for tirzepatide.

### 5.1. Lifestyle Interventions

Caloric restriction and structured exercise reduce visceral adipose tissue and improve renal outcomes, including albuminuria regression [78]. A meta-analysis of 40 RCTs (2190 participants) demonstrated that both interventions reduce visceral adiposity, but only exercise shows a dose–response relationship with VAT reduction [79]. The Look AHEAD trial demonstrated that intensive lifestyle intervention in overweight/obese individuals with T2D reduced the incidence and progression of diabetic nephropathy [80]. The renal benefits are attributed to reduced glomerular hyperfiltration, improved insulin sensitivity, decreased RAS activation, attenuated inflammation, and improved adiponectin signaling [81].

### 5.2. Pharmacological Advances

Recent pharmacological advances include incretin analogues (GLP-1 RAs and dual GIP/GLP-1 Agonists), SGLT2i and non-steroidal MRAs.

#### 5.2.1. GLP-1 RAs and Dual GIP/GLP-1 Agonists

Semaglutide promotes weight loss through GLP-1 receptor activation, with the STEP programme demonstrating 14.9–15% sustained weight reduction [82]. Beyond weight loss, semaglutide exerts direct renoprotective effects by attenuating renal inflammation, oxidative stress, endothelial dysfunction, and profibrotic signaling, partly through NHE3 inhibition in the proximal tubule, restoring tubuloglomerular feedback [52,77]. The FLOW trial [83] provided the first kidney-specific evidence: among 3533 participants with T2D and CKD, semaglutide reduced major kidney disease events by 24% (HR 0.76; 95% CI 0.66–0.88) and also reduced cardiovascular events and all-cause mortality [84], with consistent benefits across CKD stages and regardless of concomitant SGLT2i or MRA use [85]. The REMODEL trial (NCT04865770) is investigating the structural renal mechanisms underlying this protection.

Tirzepatide, a dual GIP/GLP-1 agonist, achieves even greater weight reductions (20.9% at the highest dose, SURMOUNT-1 [86]) and has demonstrated superiority over semaglutide (20.2% vs. 13.7%, SURMOUNT-5 [87]). The TREASURE-CKD trial (NCT05536804) is investigating its renoprotective mechanisms.

#### 5.2.2. SGLT2 Inhibitors

SGLT2i slow CKD progression through mechanisms extending well beyond glycemic control. By blocking proximal tubular sodium-glucose reabsorption, they restore tubuloglomerular feedback, promote afferent arteriolar vasoconstriction, and reduce intraglomerular pressure [88]. The DAPA-CKD trial demonstrated a 39% reduction in the composite kidney endpoint (HR 0.61; 95% CI 0.51–0.72), regardless of diabetes status [89]. EMPA-KIDNEY confirmed these findings in a broader CKD population, with a 28% reduction in kidney disease progression or cardiovascular death (HR 0.72; 95% CI 0.64–0.82) [90]. Beyond hemodynamics, SGLT2i decrease weight and promote metabolic reprogramming: the negative energy balance reduces visceral adiposity, improves insulin sensitivity, and shifts energy metabolism toward ketone utilization [91]. Emerging evidence suggests that empagliflozin reduces renal lipid accumulation and inflammation [92], while dapagliflozin decreases epicardial adipose tissue [93].

#### 5.2.3. Non-Steroidal MRAs

MR overactivation in obesity is driven by adipocyte aldosterone production and leptin-mediated pathways, independently of the classical RAS, creating a state of chronic MR overactivation that accelerates kidney injury [94]. Finerenone provides significant anti-inflammatory and antifibrotic effects while maintaining a favorable safety profile [95]. The FIDELIO-DKD trial demonstrated an 18% reduction in the primary renal composite outcome (HR 0.82; *p* = 0.001) and a 14% reduction in cardiovascular events [96]. FIGARO-DKD showed a 13% reduction in cardiovascular composite outcomes [97], and FIND-CKD extended benefits to non-diabetic CKD with a 23% reduction in composite kidney/cardiovascular outcomes [23]. In the FIDELITY pooled analysis, finerenone was effective regardless of obesity status, with greater benefits in patients with increased waist circumference [98]. While no clinically significant impact on body weight or visceral fat has been reported [95], finerenone reduced the risk of new-onset diabetes by 24% in the FINEARTS-HF trial [99], with consistent benefits regardless of baseline insulin resistance [100], suggesting potential metabolic benefits beyond mineralocorticoid blockade.

### 5.3. Bariatric and Metabolic Surgery

Bariatric and metabolic surgery (BMS) represents the most effective intervention for sustained weight loss in severe obesity, with renoprotective effects attributed not only to weight reduction but also to the marked decrease in VAT and associated metabolic, inflammatory, and hemodynamic improvements [101]. The SOS study demonstrated sustained reductions in albuminuria, remission from microalbuminuria to normoalbuminuria, and slower eGFR decline over 15 years of follow-up [102]. Multiple cohort studies [103] and meta-analyses confirm significant reductions in albuminuria, proteinuria, and glomerular hyperfiltration [104]. Importantly, a meta-analysis showed that BMS improves renal outcomes independently of total weight reduction, supporting the concept of metabolic rather than purely weight-based intervention [105]. Mechanisms include improved insulin sensitivity, modified incretin secretion, reduced RAS activation, and decreased pro-inflammatory adipokines [106]. The marked VAT reduction correlates more closely with renal improvement than overall weight loss [66,107].

BMS is particularly beneficial in obese individuals with T2D, where diabetes remission attenuates hyperglycemia-driven renal injury. BMS significantly lowered MACE risk over 8 years versus medical care [108]. Direct comparisons show greater weight loss with surgery versus GLP-1 RAs (28.3% vs. 10.3%) [109], and randomized trials confirm superiority in inducing albuminuria remission [110]. BMS is relatively safe in CKD patients, though renal adverse events (AKI, nephrolithiasis, rare oxalate nephropathy) can occur [111,112].

### 5.4. Combination Therapy: GLP-1 RAs and SGLT2i

The combination of GLP-1 RAs and SGLT2i is emerging as a promising strategy for ob-CKD, though robust randomized evidence is still lacking, particularly in non-diabetic CKD. Current data from trials such as DECLARE-TIMI 58 and EMPA-REG OUTCOME show consistent renal protection across BMI categories [113,114]. A recent meta-analysis demonstrated that long-term SGLT2i use is associated with significant reductions in fat mass and body weight while preserving fat-free mass [115], suggesting a more favorable body composition effect than initially appreciated . Post hoc analyses from FLOW and real-world cohorts suggest additive benefits, including greater reductions in weight, HbA1c, albuminuria, and improved eGFR trajectories, with no safety concerns [116,117]. However, no dedicated sequencing or head-to-head trials exist, and optimal use of combination therapy remains to be defined.

Non-steroidal MRAs represent a complementary pillar in multimodal therapy. A prespecified pooled participant-level analysis of FIDELIO-DKD, FIGARO-DKD, and FINEARTS-HF (FINE-HEART) demonstrated that finerenone consistently reduced cardiovascular death or heart failure hospitalization regardless of baseline BMI (interaction *p* = 0.27) or waist-to-height ratio (*p* = 0.26), with greater absolute benefits in patients with higher adiposity [118]. These data, together with the FIDELITY analysis showing enhanced efficacy in patients with increased waist circumference [98,118] and the FIND-CKD extension to non-diabetic CKD [23], with a pooled individual participant-level analysis (INFINITY) confirming consistent efficacy and safety across diabetic and non-diabetic CKD [119], support the integration of finerenone within combination strategies targeting the adipo–renal axis across the CKD spectrum.

## 6. Future Directions and Unmet Needs

Several important questions remain unanswered. First, the VAI lacks universally validated thresholds; the original equation was derived from white European adults [120], and ethnicity- and age-specific cut-offs are needed for global applicability. Table 5 summarizes available ethnicity-specific thresholds for identifying metabolic syndrome and visceral adipose dysfunction, but prospective studies validating their capacity to predict renal outcomes, cardiovascular events, and mortality are still required [121].

Second, standardized diagnostic criteria for ob-CKD remain an important unmet need. Currently, no consensus biomarker panel or histological scoring system exists to reliably differentiate ob-CKD from other nephropathies or to diagnose it as a concomitant condition when a second cause of CKD is present. The histological hallmarks (glomerulomegaly, secondary FSGS) are not pathognomonic, ectopic lipid evaluation is limited by conventional tissue processing, and clinical diagnosis relies largely on exclusion. Development of validated diagnostic criteria, including potential integration of imaging biomarkers (renal sinus fat), urinary lipidomics, and histological scoring, would strengthen clinical recognition and enable future interventional trials.

Third, long-term data on the synergistic effects of GLP-1 RAs and SGLT2i in obese patients with early-stage CKD are needed. Although the SELECT trial demonstrated that semaglutide 2.4 mg reduced major cardiovascular events by 20% and slowed kidney-function decline in overweight/obese individuals with established cardiovascular disease without requiring diabetes, including participants receiving concomitant SGLT2i [123], no trial has been specifically designed to evaluate this combination in non-diabetic CKD. Fourth, the development of novel biomarkers for early detection of renal lipotoxicity and adipose-mediated kidney injury represents an important unmet need. Emerging approaches, including lipidomics, urinary metabolomics, and machine-learning-based imaging analysis, hold promise for improved risk stratification but are not yet validated for clinical use [124]. Finally, aldosterone synthase inhibitors (baxdrostat, lorundrostat) represent an emerging pharmacological class that directly suppresses aldosterone production upstream of the mineralocorticoid receptor; preliminary data in hypertensive CKD patients are promising [125], but dedicated renal outcome trials are needed to define their role in ob-CKD.

## 7. Conclusions

The evidence reviewed converges on a central guiding concept: the adiporenal axis, the bidirectional crosstalk between dysfunctional visceral adipose tissue and the kidney, represents both the mechanistic core of ob-CKD and its most actionable therapeutic target. The progression from BMI to waist circumference to VAT as the preferred assessment metric reflects a parallel maturation in understanding: from total mass through central distribution to depot-specific dysfunction. Just as BMI misclassifies risk by ignoring fat distribution and muscle mass, and waist circumference improves but still conflates subcutaneous with visceral fat, only direct VAT quantification or validated surrogates such as the VAI capture the pathogenic compartment driving hemodynamic, inflammatory, adipokine-mediated, and lipotoxic injury.

From a therapeutic standpoint, the landscape has evolved from supportive measures to mechanism-targeted interventions that simultaneously address weight, metabolic dysfunction, and renal protection. GLP-1 RAs and dual GIP/GLP-1 agonists achieve unprecedented weight loss with direct kidney-protective mechanisms beyond metabolic improvement. SGLT2i restore tubuloglomerular feedback and reduce hyperfiltration independent of glycemic status. Non-steroidal MRAs (finerenone) address the residual inflammatory and fibrotic burden amplified by visceral fat and may improve the metabolic component. BMS remains the most effective intervention for sustained visceral fat reduction and albuminuria remission in severe obesity, with emerging evidence of benefits independent of total weight loss. Anchoring both assessment and treatment in visceral adiposity rather than BMI corrects the systematic misclassification that has historically undermined prevention, identifies high-risk individuals earlier, and aligns therapeutic intensity with the pathogenic compartment that drives kidney disease.

## Figures and Tables

**Table 1:** Epidemiological cohort studies and meta-analyses linking visceral adiposity markers to renal outcomes.

Study (PMID, Year)	Design/Population	Adiposity Marker	Renal Outcome	Main Result
[2] (39390683, 2024)	Mendelian randomization; UK Biobank, CKDGen, FinnGen	Genetically predicted VAT	CKD, eGFR(cys), BUN	VAT causally ↑ CKD (OR 1.18–1.47); hypertension mediates ~46%
[3] (37095344, 2023)	Prospective; 56 CKD patients (biopsy)	Renal sinus fat volume (CT/MRI)	>50% eGFR decline	Higher renal sinus fat → faster decline and hypertension
[63]—REGARDS (36522626, 2022)	Prospective; 27,550 adults	VAI, LAP, WC, BMI	Incident CKD (eGFR < 60 & >25% drop)	All markers ↑ incident CKD; VAI/LAP not beyond WC/BMI
[62] (40979159, 2025)	Population cohort; 9916 adults ≥ 40 y; 3.6 y	Visceral Adiposity Index (VAI)	Incident albuminuria & CKD	Top VAI quartiles: HR ≈ 1.5 for both outcomes
[59]—MESA (25210651, 2013)	Prospective; 4573 non-diabetic adults	BMI, WC, WHR	Rapid eGFR decline/incident CKD	WC and WHR associated with decline; varies by filtration marker
[65]—Oh et al. (24454716, 2014)	Prospective; 390 older adults; 5.3 y	Body-fat % change (BIA)	eGFR decline	↑ body-fat % → rapid decline, independent of BMI/WC
[64]—NHANES (38856937, 2024)	Cross-sectional; 18,078 adults	TyG index vs. VAI, LAP	CKD, albuminuria	All ↑ CKD/albuminuria; TyG best discrimination
[60] (38582736, 2024)	Meta-analysis; 13 studies, 55,175 CKD	WC/WHR vs. BMI, fat mass	All-cause mortality	High WHR ↑ mortality (HR 1.31); BMI shows paradox
[61] (39661254, 2024)	Meta-analysis; 23 studies, 381,580 HD	BMI vs. WC/WHR/BIA	All-cause mortality	BMI inverse (paradox); WC/WHR & BIA fat ↑ mortality

Abbreviations: BMI: body mass index; CKD: chronic kidney disease; eGFR: estimated glomerular filtration rate; HR: hazard ratio; LAP: lipid accumulation product; MESA: Multi-Ethnic Study of Atherosclerosis; NHANES: National Health and Nutrition Examination Survey; OR: odds ratio; REGARDS: Reasons for Geographic and Racial Differences in Stroke; TyG: triglyceride-glucose index; VAI: Visceral Adiposity Index; VAT: visceral adipose tissue; WHR: waist-to-hip ratio. Summary of key observational studies and meta-analyses examining the association between visceral adiposity measures (VAT, waist circumference, WHR, VAI, LAP, TyG index) and renal outcomes (incident CKD, albuminuria, eGFR decline, mortality). Studies include the UK Biobank, MESA, REGARDS, NHANES, and hemodialysis meta-analyses. The table reports study design, population size, adiposity metric, primary renal endpoint, and main effect estimate. ↑ indicates an increased risk or a positive association; → indicates ‘leads to’.

**Table 2:** Classification of obesity-related kidney disease (ob-CKD)

Type	Presentation	Proposed Treatment
ob-CKD type 1	Kidney functional alterations in obesity (hyperfiltration)	Non-pharmacological interventions, RAASi, SGLT2i, incretins, bariatric surgery
ob-CKD type 2	Kidney disease directly associated with obesity (slowly progressive proteinuria with variable decline in eGFR)	
ob-CKD type 3	Obesity in other primary kidney diseases	≈ob-CKD type 1 and 2 + specific treatment of primary kidney disease
ob-CKD type 4	Obesity in dialysis	Non-pharmacological interventions, incretins, bariatric surgery
ob-CKD type 5	Obesity in kidney transplant	≈ob-CKD type 1 and 2 + adjustment of immunosuppressant therapy

Abbreviations: CKD: chronic kidney disease; eGFR: estimated glomerular filtration rate; ob-CKD: obesity-related chronic kidney disease; ORG: obesity-related glomerulopathy; RAASi: renin–angiotensin–aldosterone system inhibitor; SGLT2i: sodium-glucose cotransporter 2 inhibitor. Recently proposed five-tier classification of obesity-related chronic kidney disease, encompassing: type 1 (functional alterations/hyperfiltration), type 2 (ORG with progressive proteinuria), type 3 (obesity coexisting with other primary kidney diseases), type 4 (obesity in dialysis), and type 5 (obesity in kidney transplant recipients). For each type, the table specifies clinical presentation and proposed therapeutic strategies.

**Table 3:** Clinical, histopathological, prognostic and therapeutic differences between ORG (obesity-related glomerulopathy) and primary FSGS [5].

Feature	ORG	Primary FSGS
Age at presentation	Most common in middle-aged adults, but may be present in children and older adults	Most common in children and young adults
Etiology	Obesity-induced hyperfiltration and metabolic stress	Primary podocytopathy (anti-nephrin antibodies…)
Clinical presentation	Insidious, slowly progressive	Sudden onset
Proteinuria and serum albumin	Usually subnephrotic; nephrotic range possible. Normal serum albumin levels	Typically nephrotic-range
Nephrotic syndrome	Rare (<5%)	Common
Glomerulomegaly	Universal	Uncommon (10% cases)
FSGS pattern	Predominantly perihilar	Tip, collapsing or NOS
Extent of lesions	Limited proportion of glomeruli	More widespread
Foot process effacement	Mild, segmental (<50%)	Diffuse (>50%)
Podocyte injury	Adaptive (hypertrophy → depletion)	Primary injury
Tubulointerstitial damage	Mild to moderate	Frequently severe
Metabolic abnormalities	Common (HTN, dyslipidemia, IR)	Not typical
Disease course	Slow progression	More aggressive
Renal survival	Better (≈75% at 5 years, and 50% at 10 years)	Worse (≈50% at 5 years and 25% at 10 years)
ESRD risk	10–33%	Higher
Reversibility	Partial (early weight loss)	Limited
Treatment	Weight loss, RAASi, SGLT2i, incretins, metabolic surgery	Immunosuppression + RAASi

Abbreviations: ESRD: end-stage renal disease; FSGS: focal segmental glomerulosclerosis; HTN: hypertension; IR: insulin resistance; NOS: Not Otherwise Specified; ORG: obesity-related glomerulopathy; RAASi: renin–angiotensin–aldosterone system inhibitor; SGLT2i: sodium-glucose cotransporter 2 inhibitor. Side-by-side comparison of obesity-related glomerulopathy and primary focal segmental glomerulosclerosis across 17 clinical and histopathological features, including age at presentation, etiology, proteinuria pattern, glomerular findings (glomerulomegaly, FSGS pattern, foot process effacement), metabolic associations, disease course, renal survival, and therapeutic approach.

**Table 4:** Major clinical trials evaluating nephroprotection, kidney outcomes and weight-loss efficacy in patients with obesity and metabolic disease.

Clinical Trial	Drug	Population	Renal Endpoint	Main Renal Result
FLOW	Semaglutide sc vs. placebo	3533 patients with T2D and CKD	Composite of kidney failure, sustained ≥50% decline in eGFR, or death from renal or cardiovascular causes	Semaglutide reduced the risk of major kidney outcomes by 24% compared with placebo (HR 0.76; 95% CI 0.66–0.88).
EMPA-KIDNEY	Empagliflozin 10 mg vs. placebo	6609 patients with CKD, with or without T2D	Kidney disease progression (ESKD, sustained eGFR decline, or renal death) or cardiovascular death	Empagliflozin reduced the risk of kidney disease progression or cardiovascular death by 28% (HR 0.72; 95% CI 0.64–0.82).
DAPA-CKD	Dapagliflozin 10 mg vs. placebo	4304 patients with CKD, with or without T2D	Sustained ≥50% decline in eGFR, ESKD, or death from renal or cardiovascular causes	Dapagliflozin reduced the primary composite renal outcome by 39% (HR 0.61; 95% CI 0.51–0.72).
FIDELIO-DKD	Finerenone 10/20 mg vs. placebo	5734 patients with CKD and T2D	Kidney failure, sustained ≥40% decline in eGFR, or renal death	Finerenone reduced the risk of CKD progression by 18% (HR 0.82; 95% CI 0.73–0.93).
FIGARO-DKD	Finerenone 10/20 mg vs. placebo	7437 patients with T2D and earlier-stage CKD	Secondary renal composite: kidney failure, sustained ≥40% decline in eGFR, or renal death	Non-significant trend toward renal benefit (HR 0.87; 95% CI 0.76–1.01), with stronger cardiovascular protection.
FIND-CKD	Finerenone 10/20 mg vs. placebo	1584 patients with CKD and albuminuria without T2D	Total eGFR slope (primary endpoint); secondary composite of sustained ≥57% decline in eGFR, kidney failure, hospitalization for heart failure, or cardiovascular death	Finerenone reduced annual eGFR slope difference by 0.7 mL/min/1.73 m^2^/year (*p* < 0.001) and the risk of the composite kidney–cardiovascular outcome by 23% (HR 0.77; 95% CI 0.60–0.99).
STEP 1	Semaglutide 2.4 mg sc vs. placebo + lifestyle intervention	1961 adults with overweight or obesity without diabetes	No dedicated renal endpoint	Significant weight loss (−14.9% body weight at 68 weeks); renal outcomes were not primary study endpoints.
SURMOUNT-1	Tirzepatide vs. placebo	2539 adults with obesity or overweight without diabetes	No dedicated renal endpoint	Marked weight reduction (up to −20.9% body weight at 72 weeks); renal outcomes were not evaluated as primary endpoints.

Abbreviations: CI: confidence interval; CKD: chronic kidney disease; eGFR: estimated glomerular filtration rate; ESKD: end-stage kidney disease; HR: hazard ratio; sc: subcutaneous; T2D: type 2 diabetes. Summary of pivotal randomized controlled trials assessing cardiorenal outcomes of GLP-1 receptor agonists (FLOW, STEP 1, SURMOUNT-1), SGLT2 inhibitors (DAPA-CKD, EMPA-KIDNEY), and non-steroidal mineralocorticoid receptor antagonists (FIDELIO-DKD, FIGARO-DKD, FIND-CKD). For each trial, the table reports the drug, population, renal endpoint, and main result.

**Table 5:** Visceral Adiposity Index (VAI) cut-off thresholds by ethnic group.

Ethnic Group	VAI Cut-Off Value	Primary Medical Target	Reference
White/Caucasian (European)	2.00–2.52	Metabolic Syndrome & Heart Risk	Amato MC [120]
Black/African American	1.58	Metabolic Syndrome	Di Natale JC [122]
Hispanic/Latino	1.87–1.91	Dysfunctional Adiposity	Bermudez VJ et al. [121]
East Asian/Chinese	Formula Modified (CVAI)	Visceral Fat Accumulation	Xia MF et al. [58]
Middle Eastern/Iranian	4.86	Onset type 2 Diabetes Risk	Bagheri A et al. [59]

Abbreviations: CVAI: Chinese Visceral Adiposity Index; VAI: Visceral Adiposity Index. Ethnicity-specific VAI thresholds proposed for the identification of metabolic syndrome and visceral adipose dysfunction in major global populations. Values are reported for White/Caucasian (European), Black/African American, Hispanic/Latino, East Asian/Chinese (using the modified Chinese Visceral Adiposity Index, CVAI), and Middle Eastern/Iranian populations. Age-stratified cut-offs are recommended, as younger individuals show higher predictive sensitivity at lower values.

## Data Availability

No new data were created or analyzed in this study. Data sharing is not applicable to this article.
